# Primary tumor-derived, multiparametric MRI-based deep learning-radiomics-clinical model for predicting lymph node metastasis in early-stage cervical cancer

**DOI:** 10.1186/s13244-026-02211-w

**Published:** 2026-02-09

**Authors:** Yu Hao Bao, Yan Chen, Mei Ling Xiao, Yong Ai Li, Feng Hua Ma, Hai Ming Li, Jing Yan Wu, Guo Fu Zhang, Jin Wei Qiang

**Affiliations:** 1 Department of Radiology, Jinshan Hospital, Fudan University, Shanghai, China; 2 Department of Nuclear Medicine, The First Affiliated Hospital, Zhejiang University School of Medicine, Hangzhou, China; 3 Departments of Radiology, Obstetrics & Gynecology Hospital, Fudan University, Shanghai, China; 4 Department of Radiology, Fudan University Shanghai Cancer Center, Shanghai, China

**Keywords:** Uterine cervical neoplasms, Lymphatic metastasis, Magnetic resonance imaging, Radiomics, Deep learning

## Abstract

**Objectives:**

To develop and validate a primary tumor-derived, multiparametric MRI-based deep learning-radiomics-clinical (DLRC) model for predicting pelvic lymph node metastasis (LNM) in early-stage cervical cancer.

**Materials and methods:**

This retrospective five-center study selected 1095 patients (Jan 2020-Dec 2022), divided into training (*n* = 481), internal validation (*n* = 204), and external validation (*n* = 410) cohorts. Radiomics and deep learning (DL) features were extracted from the volumetric segmentations of the primary cervical tumors on three MRI sequences (CE-T1WI, DWI, FS-T2WI). After constructing individual radiomics and DL models, the DLRC model was developed by integrating the radiomics_score, optimal DL model, and significant clinical features. Model performance was evaluated using ROC analysis, calibration curves, and decision curve analysis.

**Results:**

The DLRC model demonstrated superior predictive performance, achieving AUCs of 0.807 (95% CI: 0.766–0.849) in the training cohort, 0.789 (95% CI: 0.721–0.857) in the internal validation cohort, and 0.807 (95% CI: 0.761–0.853) in the external validation cohort. It significantly outperformed both the radiomics model and the optimal DL model (all *p* < 0.001) in the external validation cohort. The calibration curves indicated good agreement between predictions and observations. The decision curve analysis showed that the DLRC model provided the highest net clinical benefit across most decision thresholds.

**Conclusions:**

The DLRC model, which integrates tumor-derived multiparametric MRI features with clinical features, represents a robust and generalizable tool for the preoperative prediction of LNM. Its comparable accuracy to standardized radiological assessment and clinical utility shows potential to aid in personalized treatment planning for patients with early-stage cervical cancer.

**Critical relevance statement:**

The combined model (DLRC) integrating deep learning and radiomics features from the primary tumor with clinical characteristics enables preoperative LNM risk stratification, supporting personalized surgical planning and reducing unnecessary lymphadenectomy.

**Key Points:**

- Accurate preoperative prediction of lymph node metastasis in early-stage cervical cancer remains a significant clinical challenge.
- The model integrating deep learning and radiomics features derived from the primary tumor with clinical features achieved robust and generalizable predictive performance.

- The accuracy of a deep learning-radiomics-clinical nomogram for lymph node metastasis risk stratification in early-stage cervical cancer is comparable to standardized radiological assessment.

**Graphical Abstract:**

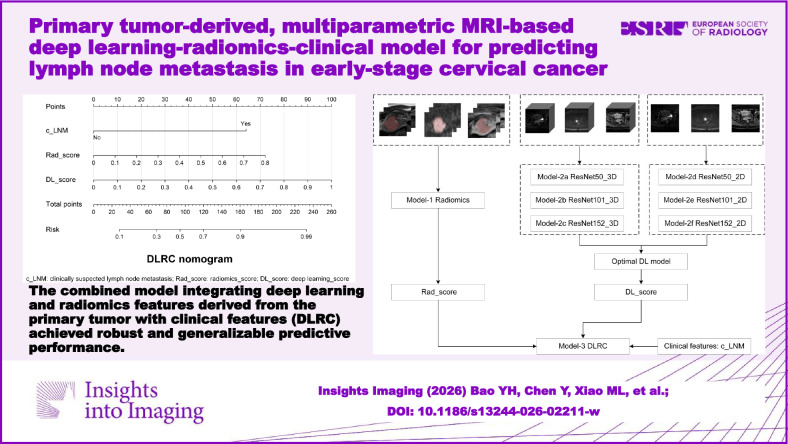

## Introduction

Cervical cancer is the fourth most common malignancy among women, with approximately 600,000 new cases and 340,000 deaths reported globally in 2020 [1]. The standard treatment for early-stage cervical cancer includes radical hysterectomy with pelvic lymphadenectomy. Although surgical intervention achieves favorable 5-year survival outcomes, the prognosis worsens significantly when pelvic lymph node metastasis (LNM) occurs, with survival rates declining from 85–90% to 50–55% [2]. LNM is the most significant independent risk factor for both tumor recurrence and overall survival [3].

In the 2018 edition of the International Federation of Gynecology and Obstetrics (FIGO) staging system for cervical cancer, imaging techniques were incorporated for the first time to assess LNM status [4]. Once LNM is diagnosed, the FIGO stage is uniformly upgraded to IIIC. Treatment selection primarily depends on the preoperative clinical FIGO stage. According to the National Comprehensive Cancer Network guidelines, early-stage cervical cancer (stage IIA and below) is treated with radical surgery, while concurrent chemoradiotherapy is recommended for advanced-stage disease [5].

Accurate preoperative assessment of LNM is critical for optimizing individualized treatment strategies. However, clinical staging is not always accurate [6], as approximately 10–30% of patients with early-stage cervical cancer have LNM [7], suggesting that a significant proportion of patients may not benefit directly from lymphadenectomy and are at risk of intraoperative and postoperative complications, including hemorrhage, infection, lymphocele, and lower limb lymphedema [8].

Histopathological examination remains the gold standard for assessing LNM. However, it is an invasive procedure and is only available postoperatively. MRI is widely employed for the preoperative evaluation of LNM, with the lymph node short-axis diameter (LNSD) > 10 mm commonly used as the diagnostic threshold. However, the diagnostic performance of this criterion is suboptimal. A meta-analysis reported a pooled sensitivity of only 47% for LNM detection when applying this cutoff. Another meta-analysis demonstrated sensitivities of 57% (95% CI: 0.49–0.64) for MRI and 57% (95% CI: 0.48–0.65) for PET/CT [9, 10]. However, PET/CT is costly, not widely available, and involves radiation exposure, which limits its widespread use. Therefore, a non-invasive, reliable, and cost-effective approach for LNM evaluation is urgently needed.

Previous studies have successfully applied MRI-based radiomics models to predict LNM in cervical cancer [11–13]. However, most studies were limited by small sample sizes (*n* < 200) and the absence of external validation. Deep learning (DL) has demonstrated outstanding performance in image analysis by automatically extracting high-level features from medical images [14]. It is worth trying to explore LNM by combining DL with radiomics based on multiparametric-MRI (mp-MRI).

This study aims to develop a primary tumor-derived, mp-MRI-based fusion model integrating DL, radiomics, and clinical features (DLRC) for predicting LNM in patients with early-stage cervical cancer.

## Materials and methods

### Patient data

**Fig. 1:**
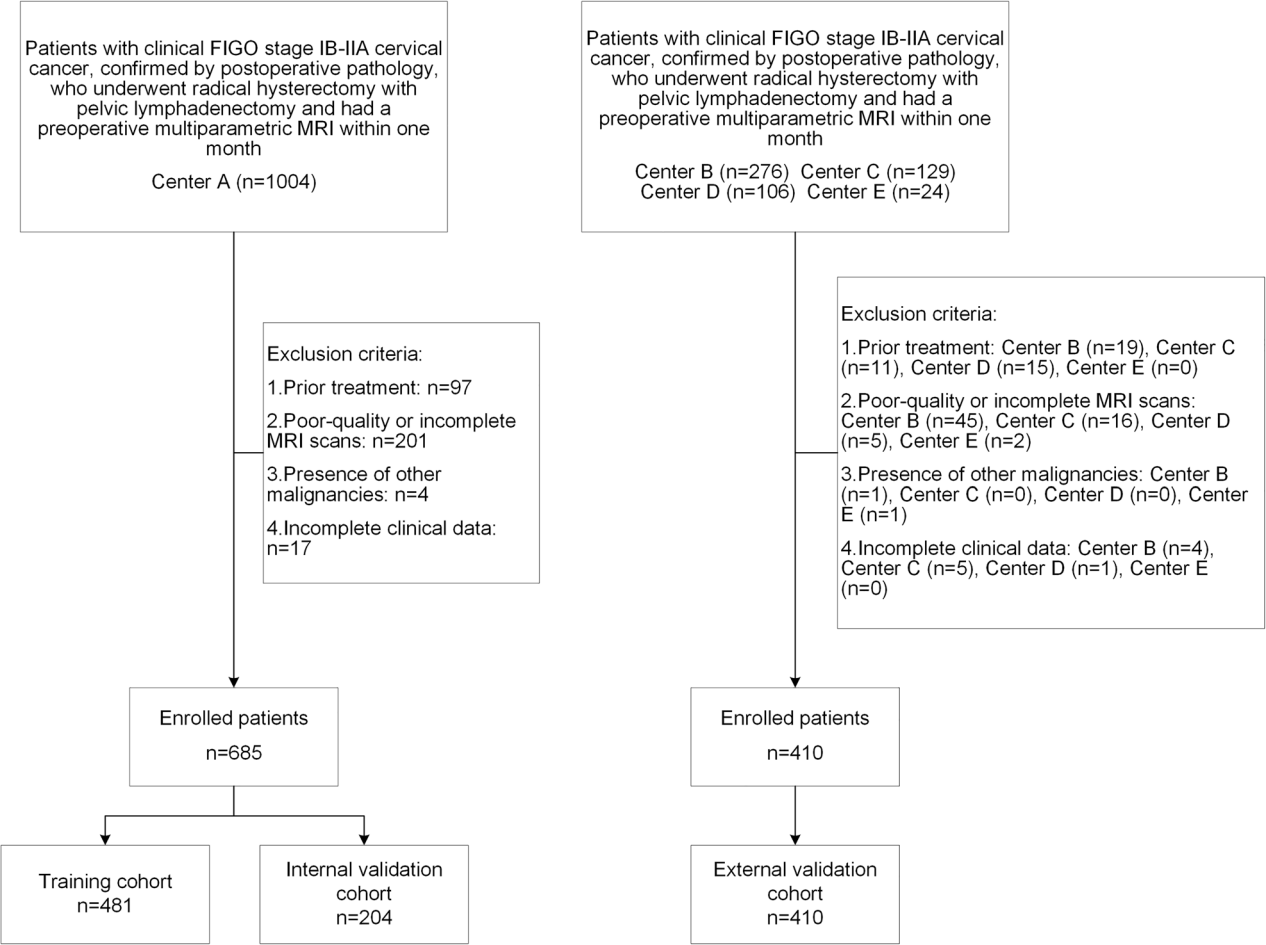
Flowchart of inclusion and exclusion criteria and grouping for early-stage cervical cancer cases from five centers

This retrospective study, approved by the institutional review board of Jinshan Hospital, Fudan University, with a waiver of patient consent (approval number: JIEC 2024-S30), enrolled 1539 patients with early-stage cervical cancer from five centers: A, C, D, E (January 2020–December 2022) and B (January 2022–December 2022). Inclusion criteria were: (1) Underwent radical hysterectomy with pelvic lymphadenectomy; (2) preoperative MRI performed within 1 month prior to surgery, including axial fat-saturated T2-weighted imaging (FS-T2WI), contrast-enhanced T1-weighted imaging (CE-T1WI), and diffusion-weighted imaging (DWI); (3) clinical FIGO stage IB–IIA cervical cancer; (4) postoperative histopathology confirming cervical cancer and lymph node status. Exclusion criteria were: (1) presurgical treatment (e.g., neoadjuvant chemoradiotherapy); (2) poor-quality or incomplete MRI scans; (3) presence of other malignancies; (4) incomplete clinical data. The inclusion and exclusion criteria, as well as the study grouping process, are illustrated in Fig. 1.

A total of 685 cervical cancer patients from Center A were included and randomly divided into a training cohort (*n* = 481) and an internal validation cohort (*n* = 204) at a ratio of approximately 7:3. Patients from Centers B, C, D, and E (*n* = 207, 97, 85, and 21, respectively) were combined to form an external validation cohort (*n* = 410).

Clinical and pathological information was collected from medical records, including age, reproductive history, menstrual history, family history of malignancies, clinical FIGO stage, tumor histological subtype, and pathological LNM status.

The standardized radiological assessment (SRA) for diagnosing LNM was defined by the following criteria: (1) pelvic LNSD ≥ 10 mm; (2) presence of central necrosis in the lymph node [15]; (3) LNSD ≥ 8 mm with a short-to-long axis ratio greater than 0.8 [16]. Radiologists 1 and 2 (Y.H.B. and Y.A.L., 5 and 11 years of experience in pelvic MRI diagnosis, respectively) independently assessed. Pelvic LNSD was measured on CE-T1WI sequences, which were selected for measurement due to their larger matrix size and the thinnest slice thickness. For each patient, the largest lymph node in terms of short-axis diameter was identified and measured. Pelvic LNSD ≥ 10 mm is defined as clinically suspected lymph node metastasis (c_LNM), which was collected as a binary clinical feature for subsequent analysis. Any discrepancies were resolved by a senior radiologist (J.W.Q., with 20 years of experience) to achieve a final consensus reading. The radiologists were aware of the cervical cancer diagnosis but were blinded to clinical details and LNM status. The detailed measurement process and results were described in Supplementary Method S1.

### MRI scanning and image segmentation

Axial FS-T2WI, DWI (b-value of 800 or 1000 s/mm²), and CE-T1WI images were collected. The scanning protocols and detailed parameters of the 12 MRI scanners are provided in Supplementary Method S2 and Table S1.

The open-source software ITK-SNAP (version 3.8.0; www.itksnap.org) was used for manual segmentation of the region of interest (ROI) slice by slice along the tumor boundaries on three sequences, while avoiding areas of hemorrhage and necrosis. This was done to focus segmentation on the biologically active, solid tumor component, as hemorrhage exhibits dynamic signal changes over time, and necrotic tissue does not contribute to metastatic potential. For 30 randomly selected cases, Radiologists 1 and 2 independently delineated the ROIs to assess the inter-observer consistency of features. Radiologist 1 repeated the ROI delineation and feature extraction for the 30 cases after a 2-month interval to evaluate the intra-observer consistency. Features with ICCs > 0.75 were retained. Radiologist 1 segmented the remaining cases. The software automatically generated the volume of interest (VOI) for radiomics feature extraction (Supplementary Fig. S1).

### Radiomics and DL feature extraction

Radiomics features were extracted from three raw segmented image sequences and their filtered versions by PyRadiomics (version 3.1.0, www.radiomics.io/pyradiomics.html) [17].

Pre-trained models of ResNet50, ResNet101, and ResNet152 [18] on ImageNet [19] were utilized. ImageNet is a large-scale image database containing over 14 million annotated images across 22,000 categories.

**Fig. 2:**
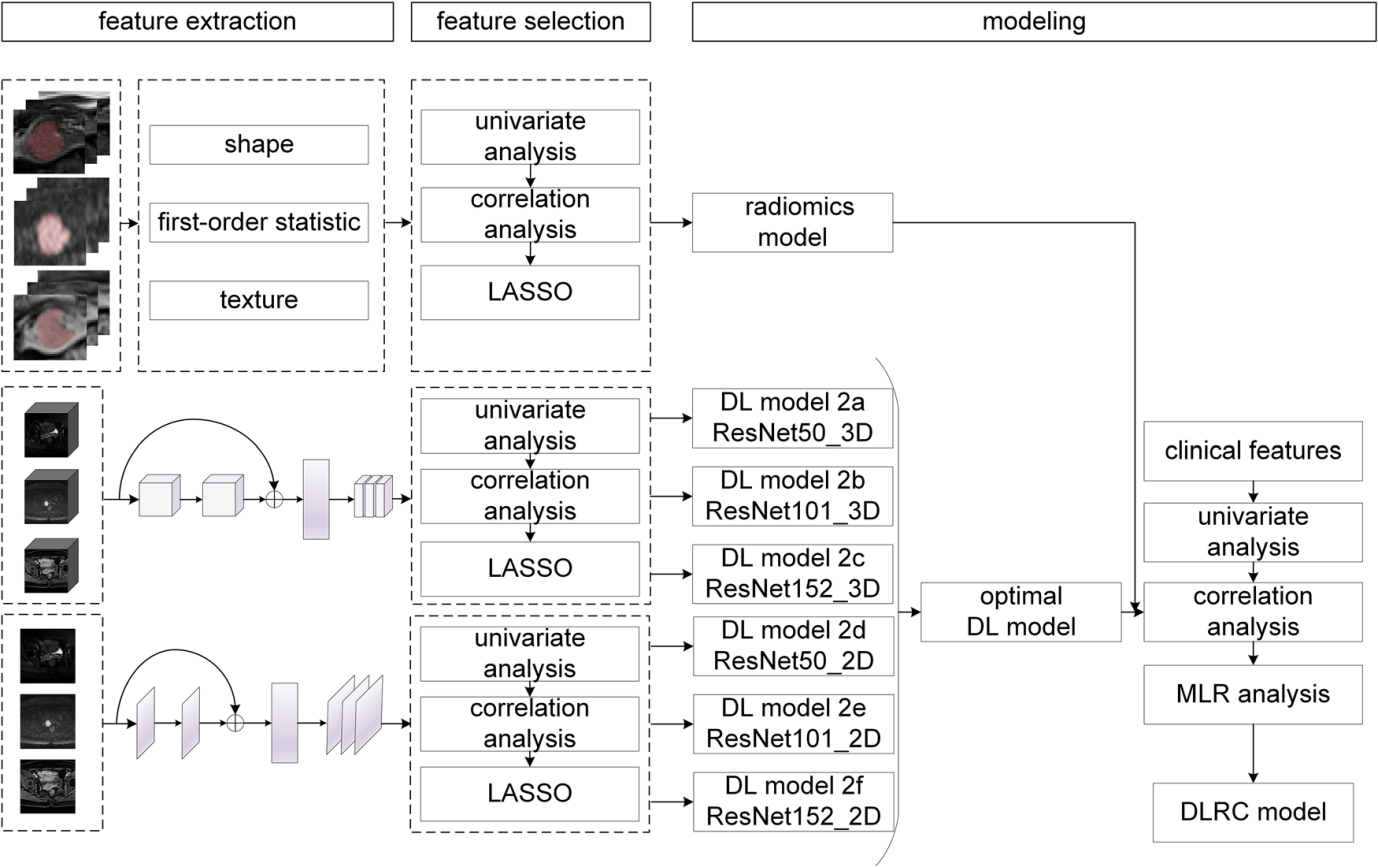
Technical workflow for constructing the lymph node metastasis (LNM) prediction models in early-stage cervical cancer. LASSO, least absolute shrinkage and selection operator; DL, deep learning; DLRC, deep learning-radiomics-clinical; MLR, multivariable logistic regression

Features were extracted based on both two-dimensional (2D) and three-dimensional (3D) images, respectively. Details of feature extraction and preprocessing can be found in the Supplementary Method S3. The technical workflow for model construction is illustrated in Fig. 2.

The ComBat method was employed to harmonize radiomics and DL features, mitigating the effects of variations in MRI scanners and scanning protocols across different centers [20]. Principal component analysis scatterplots of radiomics and DL features before and after ComBat harmonization are provided in Supplementary Figs. S2 and S3.

### Features selection and model construction

Radiomics feature selection was conducted in the training cohort. The selection and modeling process followed these steps: (1) Univariate analysis, the Mann–Whitney U test, was conducted with retaining features with *p* < 0.01; (2) Spearman correlation analysis was conducted to remove features with a correlation coefficient greater than 0.8. (3) The least absolute shrinkage and selection operator (LASSO) was applied to select features, compressing some feature coefficients to zero and retaining features with non-zero coefficients that were strongly associated with LNM. (4) Stepwise multivariable logistic regression analysis was performed, and the backward selection method based on the Akaike information criterion (AIC) score was used to determine the optimal combination of features, thereby completing the construction of the radiomics model.

Three 3D DL models, 2a (ResNet50_3D), 2b (ResNet101_3D), and 2c (ResNet152_3D), and three 2D DL models, 2d (ResNet50_2D), 2e (ResNet101_2D), and 2f (ResNet152_2D) were constructed. The feature selection and model construction process followed the same workflow as the radiomics model.

Steps for constructing the DLRC model: (1) Clinical features with *p* < 0.05 after univariate analysis were retained. (2) The best-performing DL model among the six DL models, the radiomics model, and the retained clinical features were integrated to complete the DLRC model construction by stepwise multivariable logistic regression.

### Model performance evaluation

The diagnostic performance of three models was evaluated in the training, internal validation, and external validation cohorts using receiver operating characteristic (ROC) analysis. Calibration curves were plotted based on the Hosmer-Lemeshow test to assess the agreement between predicted and observed LNM outcomes. Decision curve analysis (DCA) was used to evaluate the clinical benefit.

### Statistical analysis

All statistical analyses were performed using SPSS (version 27.0.0.0, Armonk) and R (version 4.2.1, https://www.r-project.org/). For clinical features, categorical variables were analyzed using the chi-square test or Fisher’s exact test, with Fisher’s exact test used when expected frequencies in any cell of the contingency table were less than 5. Continuous variables were assessed using either the independent samples *t*-test or the Mann–Whitney U test, depending on whether they followed a normal distribution. Reproducibility analysis was performed using ICCs. Model performance was evaluated using the ROC curve. The optimal cutoff value was determined based on the maximum Youden index (sensitivity + specificity − 1) derived from the ROC curve, and the model’s accuracy, sensitivity, specificity, negative predictive value (NPV), and positive predictive value (PPV) were calculated. The DeLong test was used to compare the AUC values among the three models. A two-tailed *p* < 0.05 was considered statistically significant.

## Results

### Clinical characteristics

The comparison of clinicopathological characteristics between the LNM-positive and LNM-negative subgroups in the training (*n* = 481), internal validation (*n* = 204), and external validation cohorts (*n* = 410) is presented in Table 1. The number of patients with LNM-positive in the training, internal validation, and external validation cohorts was 149 (30.9%), 63 (30.8%), and 127 (30.9%), respectively, with no statistically significant difference in LNM positivity rates among the three cohorts (*p* = 1.000). In the training cohort, statistically significant differences were observed in clinical FIGO staging (*p* < 0.001) and c_LNM (*p* < 0.001).

**Table 1:** Comparison of clinical characteristics between LNM-negative and LNM-positive groups in the training, internal validation, and external validation cohorts

Clinical feature	Training		*p*-value	Internal validation		*p*-value	External validation		*p*-value
	LNM(−)	LNM(+)		LNM(−)	LNM(+)		LNM(−)	LNM(+)	
	*N* = 332	*N* = 149		*N* = 141	*N* = 63		*N* = 283	*N* = 127	
Age	53.0 [43.0; 59.0]	52.0 [44.0; 59.0]	0.596	54.0 [47.0; 60.0]	54.0 [46.0; 62.0]	0.735	54.0 [46.5; 63.0]	52.0 [44.0; 58.5]	0.039
Parity			0.715			0.019			0.505
0	12 (3.6%)	8 (5.4%)		4 (2.8%)	4 (6.3%)		13 (4.6%)	4 (3.1%)	
1–2	270 (81.3%)	116 (77.9%)		103 (73.0%)	54 (85.7%)		239 (84.5%)	103 (81.1%)	
3–4	43 (13.0%)	21 (14.1%)		26 (18.4%)	5 (7.9%)		27 (9.5%)	17 (13.4%)	
≥ 5	7 (2.1%)	4 (2.7%)		8 (5.7%)	0 (0.0%)		4 (1.4%)	3 (2.4%)	
Menopause			0.349			0.565			0.079
No	148 (44.6%)	74 (49.7%)		55 (39.0%)	28 (44.4%)		106 (37.5%)	60 (47.2%)	
Yes	184 (55.4%)	75 (50.3%)		86 (61.0%)	35 (55.6%)		177 (62.5%)	67 (52.8%)	
Age at menopause	50.0 [48.0; 52.0]	50.0 [48.0; 52.0]	0.641	50.0 [49.0; 52.8]	51.0 [50.0; 53.0]	0.120	50.0 [48.0; 53.0]	51.0 [48.0; 54.0]	0.397
Family history			0.931			0.994			0.146
No	290 (87.3%)	129 (86.6%)		127 (90.1%)	56 (88.9%)		237 (83.7%)	98 (77.2%)	
Yes	42 (12.7%)	20 (13.4%)		14 (9.9%)	7 (11.1%)		46 (16.3%)	29 (22.8%)	
FIGO stage			< 0.001			0.002			0.002
ⅠB1	36 (10.8%)	4 (2.7%)		22 (15.6%)	3 (4.8%)		33 (11.7%)	5 (3.9%)	
ⅠB2	117 (35.2%)	38 (25.5%)		49 (34.8%)	13 (20.6%)		85 (30.0%)	35 (27.6%)	
ⅠB3	25 (7.5%)	24 (16.1%)		6 (4.3%)	9 (14.3%)		17 (6.0%)	12 (9.4%)	
ⅡA1	97 (29.2%)	37 (24.8%)		34 (24.1%)	15 (23.8%)		90 (31.8%)	30 (23.6%)	
ⅡA2	57 (17.2%)	46 (30.9%)		30 (21.3%)	23 (36.5%)		58 (20.5%)	45 (35.4%)	
c_LNM			< 0.001			< 0.001			< 0.001
No	324 (97.6%)	109 (73.2%)		136 (96.5%)	48 (76.2%)		275 (97.2%)	90 (70.9%)	
Yes	8 (2.4%)	40 (26.8%)		5 (3.5%)	15 (23.8%)		8 (2.8%)	37 (29.1%)	
Histological subtype			0.775			0.028			0.347
SCC	250 (75.3%)	116 (77.9%)		124 (87.9%)	46 (73.0%)		233 (82.3%)	112 (88.2%)	
AC	37 (11.1%)	12 (8.1%)		8 (5.7%)	9 (14.3%)		27 (9.5%)	11 (8.7%)	
ASC	43 (13.0%)	20 (13.4%)		9 (6.4%)	8 (12.7%)		16 (5.7%)	3 (2.4%)	
Other	2 (0.6%)	1 (0.7%)		0 (0.0%)	0 (0.0%)		7 (2.5%)	1 (0.8%)	

*LNM(−)* lymph node metastasis-negative, *LNM(+)* lymph node metastasis-positive, *c_LNM* clinically suspected lymph node metastasis, *FIGO* International Federation of Gynecology and Obstetrics, *SCC* squamous cell carcinoma, *AC* adenocarcinoma, *ASC* adenosquamous carcinoma

### Diagnostic performance of different models

The radiomics model achieved AUC values of 0.735 (95% confidence interval (CI), 0.688–0.782) in the training cohort, 0.767 (95% CI, 0.700–0.834) in the internal validation cohort, and 0.718 (95% CI, 0.665–0.771) in the external validation cohort. The performance of the model in predicting LNM is detailed in Table 2. Feature distributions and correlations are illustrated in Supplementary Figs. S4 (violin plots) and S5 (heatmap), respectively.

**Table 2:** Performance of the standardized radiological assessment, radiomics, ResNet152_2D, and DLRC models in predicting LNM in the training, internal validation, and external validation cohorts

Cohort	Model/SRA	AUC (95% CI)	ACC	SEN	SPE	PPV	NPV
Training	Radiomics	0.735 (0.688–0.782)	69.4%	59.7%	73.8%	50.6%	80.3%
	ResNet152_2D	0.751 (0.706–0.797)	64.7%	79.2%	58.1%	45.9%	86.2%
	DLRC	0.807 (0.766–0.849)	78.0%	60.4%	85.8%	65.7%	82.8%
	SRA	/	77.3%	33.6%	97.0%	83.3%	76.5%
Internal validation	Radiomics	0.767 (0.700–0.834)	71.6%	60.3%	76.6%	53.5%	81.2%
	ResNet152_2D	0.725 (0.649–0.801)	62.3%	73.0%	57.4%	43.4%	82.7%
	DLRC	0.789 (0.721–0.857)	77.0%	57.1%	85.8%	64.3%	81.8%
	SRA	/	76.0%	33.3%	95.0%	75.0%	76.1%
External validation	Radiomics	0.718 (0.665–0.771)	70.2%	60.6%	74.6%	51.7%	80.8%
	ResNet152_2D	0.716 (0.664–0.768)	63.7%	69.3%	61.1%	44.4%	81.6%
	DLRC	0.807 (0.761–0.853)	78.8%	59.8%	87.3%	67.9%	82.9%
	SRA	/	77.8%	37.8%	95.8%	80.0%	77.4%

*SRA* standardized radiological assessment, *DLRC* deep learning-radiomics-clinical, *AUC* area under the curve, *CI* confidence interval, *ACC* accuracy, *SEN* sensitivity, *SPE* specificity, *PPV* positive predictive value, *NPV* negative predictive value

The performance of six DL models in predicting LNM is detailed in Table 3. By comparing the AUC values of the six DL models across the three cohorts, Model 2f (ResNet152_2D) achieved the highest AUC values in both the training cohort (AUC = 0.751 (95% CI, 0.706–0.797)) and the external validation cohort (AUC = 0.716 (95% CI, 0.664–0.768)) among the six DL models, while also demonstrating relatively high AUC values in the internal validation cohort (AUC = 0.725 (95% CI, 0.649–0.801)). The performance of this model remained stable across all three cohorts. The violin plots of the retained feature distributions and the correlation heatmaps for the ResNet152_2D model are shown in Supplementary Figs. S6 and S7, respectively.

**Table 3:** Performance of six DL models in predicting LNM in the training, internal validation, and external validation cohorts

Cohort	DL model	AUC (95% CI)	ACC	SEN	SPE	PPV	NPV
Training	ResNet50_3D	0.726 (0.678–0.774)	65.3%	79.2%	59.0%	46.5%	86.3%
	ResNet101_3D	0.706 (0.657–0.755)	63.6%	74.5%	58.7%	44.8%	83.7%
	ResNet152_3D	0.696 (0.645–0.748)	69.2%	53.7%	76.2%	50.3%	78.6%
	ResNet50_2D	0.736 (0.688–0.784)	74.6%	51.0%	85.2%	60.8%	79.5%
	ResNet101_2D	0.744 (0.697–0.790)	64.4%	81.2%	56.9%	45.8%	87.1%
	ResNet152_2D	0.751 (0.706–0.797)	64.7%	79.2%	58.1%	45.9%	86.2%
Internal validation	ResNet50_3D	0.655 (0.574–0.736)	56.9%	68.3%	51.8%	38.7%	78.5%
	ResNet101_3D	0.737 (0.660–0.814)	65.7%	77.8%	60.3%	46.7%	85.9%
	ResNet152_3D	0.640 (0.556–0.724)	61.8%	50.8%	66.7%	40.5%	75.2%
	ResNet50_2D	0.757 (0.687–0.826)	73.0%	50.8%	83.0%	57.1%	79.1%
	ResNet101_2D	0.723 (0.652–0.793)	63.7%	82.5%	55.3%	45.2%	87.6%
	ResNet152_2D	0.725 (0.649–0.801)	62.3%	73.0%	57.4%	43.4%	82.7%
External validation	ResNet50_3D	0.613 (0.554–0.672)	53.9%	64.6%	49.1%	36.3%	75.5%
	ResNet101_3D	0.589 (0.526–0.651)	53.7%	61.4%	50.2%	35.6%	74.3%
	ResNet152_3D	0.554 (0.495–0.614)	58.5%	43.3%	65.4%	35.9%	72.0%
	ResNet50_2D	0.675 (0.616–0.733)	71.5%	44.1%	83.7%	54.9%	76.9%
	ResNet101_2D	0.671 (0.617–0.726)	57.1%	70.1%	51.2%	39.2%	79.2%
	ResNet152_2D	0.716 (0.664–0.768)	63.7%	69.3%	61.1%	44.4%	81.6%

**Fig. 3:**
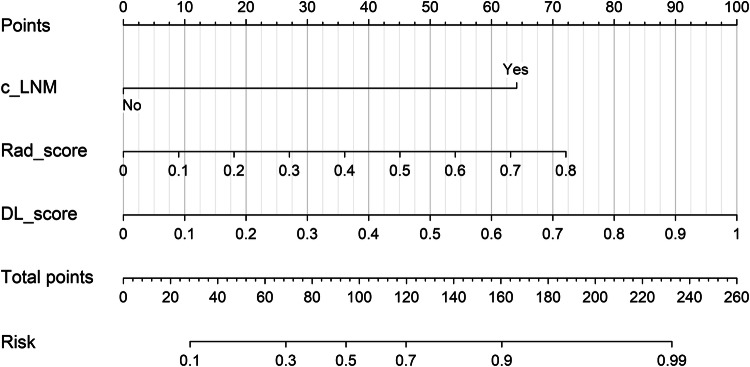
The DLRC nomogram for predicting LNM risk. To use: (1) Locate patient values on each variable axis; (2) Sum the corresponding points; (3) Project the total points to the risk axis to obtain the predicted probability. c_LNM, clinically suspected lymph node metastasis; DL_score, deep learning_score; Rad_score, radiomics_score

Clinical FIGO staging was excluded due to its inferior accuracy compared to postoperative pathological staging. ResNet152_2D, the radiomics model, and c_LNM were integrated to complete the DLRC model construction. The DLRC model achieved AUC values of 0.807 (95% CI, 0.766–0.849) in the training cohort, 0.789 (95% CI, 0.721–0.857) in the internal validation cohort, and 0.807 (95% CI, 0.761–0.853) in the external validation cohort. The performance of the DLRC model is detailed in Table 2. Figure 3 presents the DLRC nomogram, which provides a practical tool for individualized LNM risk estimation. To use it, locate patient values for each variable (c_LNM, DL_score, radiomics_score (Rad_score)) on their respective axes, sum the corresponding points to get the total score, and then read the predicted LNM risk from the bottom axis.

**Fig. 4:**
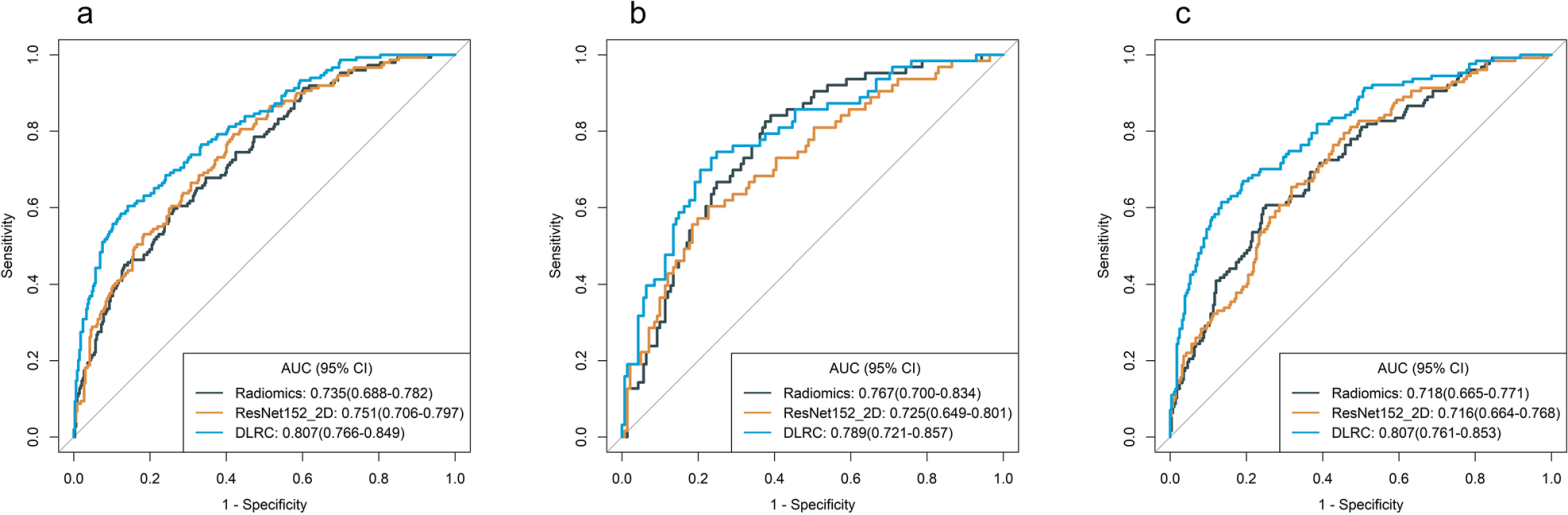
ROC curves of different models for predicting LNM in the training, internal validation, and external validation cohorts. **a**–**c** ROC curves of models in the training, internal validation and external validation cohorts, respectively. AUC, area under the curve; CI, confidence interval

The features were selected for the model construction summarized in Supplementary Table S2. The ROC curves for the radiomics, ResNet152_2D, and DLRC models are shown in Fig. 4. The DLRC model achieved the highest AUC values in all three cohorts. In the training cohort, the DLRC model significantly outperformed both the radiomics model (*p* < 0.001) and the ResNet152_2D model (*p* < 0.001). In the internal validation cohort, the DLRC model demonstrated significantly better performance than the ResNet152_2D model (*p* = 0.009), but there was no significant difference compared to the radiomics model (*p* = 0.312). In the external validation cohort, the DLRC model significantly outperformed both the radiomics model (*p* < 0.001) and the ResNet152_2D model (*p* < 0.001). There were no significant differences in LNM prediction performance between the radiomics and ResNet152_2D models in the training cohort (*p* = 0.542), the internal validation cohort (*p* = 0.260), or the external validation cohort (*p* = 0.956) (Table 4).

**Table 4:** Comparison of performance among the DLRC, radiomics, and DL models in predicting LNM in the training, internal validation, and external validation cohorts

Model comparison				*p*-value	
			Training	Inter-valid	Exter-valid
DLRC	VS	Radiomics	< 0.001	0.312	< 0.001
DLRC	VS	ResNet152_2D	< 0.001	0.009	< 0.001
Radiomics	VS	ResNet152_2D	0.524	0.260	0.956

**Fig. 5:**
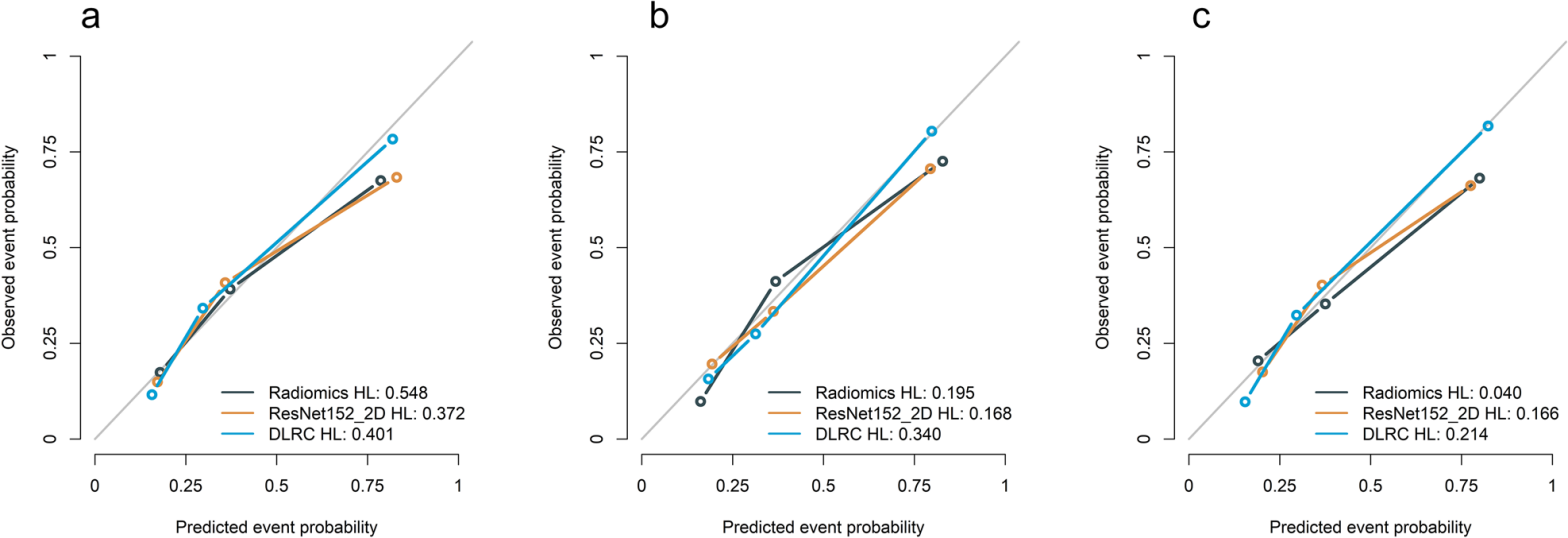
Hosmer–Lemeshow curves of different models in the training, internal validation, and external validation cohorts. **a**–**c** The calibration curves of models in the training, internal validation and external validation cohorts, respectively. The predicted event probability represents the model-estimated likelihood of LNM, while the observed event probability corresponds to the actual LNM incidence confirmed by histopathological examination

The calibration curves (Fig. 5) indicated that the DLRC model showed good agreement between predicted LNM probabilities and observed probabilities in the training cohort (*p* = 0.401), the internal validation cohort (*p* = 0.340), and the external validation cohort (*p* = 0.214), suggesting excellent model fit.

**Fig. 6:**
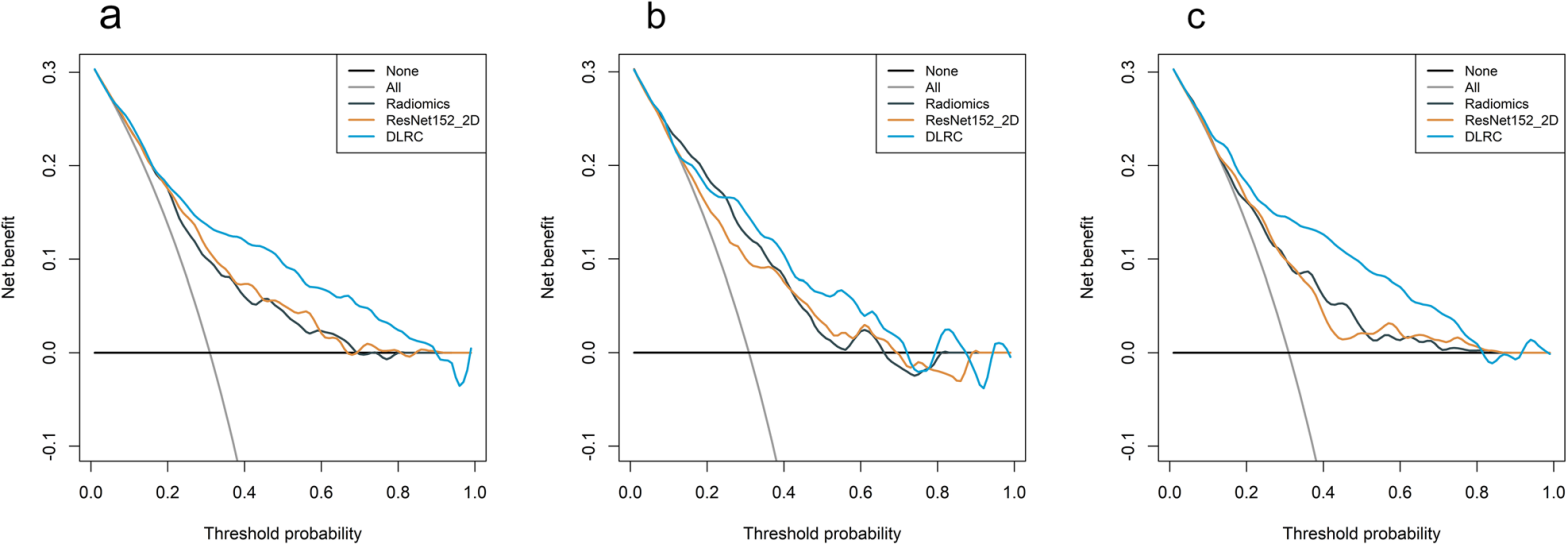
Decision curves of different models in the training, internal validation, and external validation cohorts. **a**–**c** Decision curve analysis curves of models in the training, internal validation and external validation cohorts, respectively. “None”: representing the strategy of no surgical treatment for all patients; “All”: representing the strategy of surgical treatment for all patients

The decision curve analysis (Fig. 6) demonstrated that the DLRC model provided substantial clinical benefit. Across the three cohorts, the DLRC model achieved the highest net clinical benefit over a wide range of risk thresholds.

### Comparison with SRA

The diagnostic performance of SRA in the three cohorts is summarized in Table 2. Compared to the diagnostic results of SRA, the DLRC model demonstrated a significant improvement in sensitivity. Sensitivity increased from 33.6% to 60.4% in the training cohort, from 33.3% to 57.1% in the internal validation cohort, and from 37.8% to 59.8% in the external validation cohort. While the diagnostic accuracy of the DLRC model was comparable to that of the SRA across all three cohorts, the specificity of the DLRC model was 8.5 to 11.2 percentage points lower than the latter. Subgroup analysis was performed using a 10 mm cutoff for the largest pelvic LNSD. In the LNSD < 10 mm subgroup, the DLRC model achieved a sensitivity of 43.3% in the external validation cohort, representing an absolute increase of over 30 percentage points compared to the SRA (12.2%), while maintaining comparable accuracy. Performance was largely equivalent between the DLRC model and the standardized assessment in the LNSD ≥ 10 mm subgroup (Table 5).

**Table 5:** Diagnostic performance of the DLRC model and standardized radiological assessment stratified by LNSD in the training, internal validation, and external validation cohorts

Subcohort		Model/SRA	ACC	SEN	SPE	PPV	NPV
LNSD < 10 mm	Training	DLRC	77.4%	45.9%	88.0%	56.2%	82.8%
		SRA	76.7%	9.2%	99.4%	83.3%	76.5%
	Internal	DLRC	77.2%	43.8%	89.0%	58.3%	81.8%
	Validation	SRA	76.1%	12.5%	98.5%	75.0%	76.1%
	External	DLRC	78.1%	43.3%	89.5%	57.4%	82.8%
	Validation	SRA	77.3%	12.2%	98.5%	73.3%	77.4%
LNSD ≥ 10 mm	Training	DLRC	83.3%	100.0%	0.0%	83.3%	0/0
		SRA	83.3%	100.0%	0.0%	83.3%	0/0
	Internal	DLRC	75.0%	100.0%	0.0%	75.0%	0/0
	Validation	SRA	75.0%	100.0%	0.0%	75.0%	0/0
	External	DLRC	84.4%	100.0%	12.5%	84.1%	100.0%
	Validation	SRA	82.2%	100.0%	0.0%	82.2%	0/0

## Discussion

Our study successfully developed and validated a DLRC model that, by integrating DL, radiomics, and clinical features from multiparametric MRI, demonstrated robust and generalizable performance for preoperatively predicting LNM in early-stage cervical cancer, holding promise as a decision-support tool. Accurate assessment of LNM status is crucial for early-stage cervical cancer patients, as it directly influences treatment decisions. Gynecological examinations do not provide information on LNM. Imaging modalities such as CT, PET/CT, and MRI offer more detailed disease information than gynecological exams and can clearly visualize pelvic lymph nodes. Clinically, a pelvic LNSD of 10 mm on MRI is often used as a threshold for LNM assessment. However, histopathological analysis by Williams et al [21]. demonstrated that among 504 lymph nodes from gynecological cancer patients, metastatic lesions were frequently identified in small-diameter nodes, with 54.5% of LNSD < 10 mm.

Previous studies exploring radiomics or DL for predicting LNM in cervical cancer have been limited by small external validation cohorts (11–141 patients) [22–26]. Our study, to our knowledge, represents the largest multicenter investigation to date (encompassing 1095 patients from five centers) that incorporates both internal and external validation, strongly supporting its generalizability. Furthermore, a distinctive feature of our DLRC model is its integration of DL and radiomics features derived from the primary tumor with the conventional clinical imaging characteristic c_LNM. This multiparametric approach captures complementary information: radiomics quantifies tumor heterogeneity, DL extracts sub-visual patterns, and c_LNM provides direct morphological input from the lymph nodes.

The DLRC model achieved a favorable trade-off compared to SRA, yielding a marked increase in sensitivity with only a modest reduction in specificity, while maintaining comparable overall accuracy. The consequent reduction in false negatives is critical, as the clinical impact of a missed metastasis is far more detrimental to patient survival than that of a false-positive result. Furthermore, the model’s sensitivity (59.8% in the external validation cohort) is comparable to reported values for PET/CT, while offering superior practicality through its basis in routine mp-MRI—avoiding its high cost, limited access, and radiation.

The DLRC model significantly outperformed ResNet152_2D and the radiomics model in external validation cohorts (*p* < 0.05), underscoring its superior and stable predictive performance. There were no statistically significant differences in predictive performance between the radiomics model and the optimal DL model in our study. However, previous studies have reported that DL models outperform radiomics models [27, 28]. The inconsistency between our findings and prior research suggests that further investigation is required to compare the predictive efficacy of DL and radiomics models.

Notably, for the challenging subcentimeter LNM subgroup, DLRC’s sensitivity was over 30 percentage points higher than SRA. This demonstrates the model’s incremental value through integrating primary tumor features with lymph node size, capturing metastases missed by standardized assessment (based on size, morphology, and necrosis), and highlights its clinical potential for refined risk stratification. Research has shown that using multiple imaging sequences improves diagnostic performance compared to single-sequence models. For example, Wang et al [29]. found that combining DWI and T2WI radiomics features outperformed single-sequence features, achieving an AUC of 0.909 in the internal validation cohort. Multisequence MRI provides complementary information, where DWI reflects tumor cellularity, T2WI delineates morphology, and CE-T1WI reveals vascular characteristics. These sequences are routinely available in clinical practice, facilitating the potential translation of our model. In this study, the CE-T1WI sequence demonstrated particular importance, as the features with the largest absolute coefficient value in both the radiomics (CET1_wavelet.HLH_gldm_SmallDependenceEmphasis) and optimal DL (CET1_F1695) models were derived from it. This strongly implicates tumor perfusion heterogeneity as a major factor in LNM.

Seven radiomics features, including four wavelet features, two local binary pattern features, and one gradient filtering feature, were retained for the radiomics model. Wavelet features, which reflect tumor heterogeneity and better represent image information, are major components of radiomics features and can improve outcome prediction [30]. Radiomics methods comprehensively evaluate the entire tumor by extracting imaging information from the VOI, whereas histopathological analysis of tumor biopsies provides limited tumor characteristics due to tumor heterogeneity. Compared to traditional radiomics, DL has the ability to automatically learn and prioritize key regions relevant to the research objective. Although studies have shown that DL can detect spatial tumor heterogeneity [31], the interpretability of DL models remains limited.

This study had several limitations. First, its retrospective design and recruitment from specialized centers may introduce selection bias, necessitating future prospective validation in broader settings. Second, manual segmentation, though vetted for robustness, was time- and labor-intensive (approximately 10–15 min per case), despite its demonstrated robustness, highlighting the need for automated solutions. Finally, our model focuses solely on the primary tumor, excluding lymph node segmentation, peritumoral regions, and non-viable areas (hemorrhage/necrosis), which represent valuable avenues for future research to potentially enhance performance.

## Conclusion

The DLRC model achieved the highest AUC values in the three cohorts, outperforming both the DL and radiomics models, and demonstrated comparable accuracy to SRA but higher sensitivity.

## Supplementary information

ELECTRONIC SUPPLEMENTARY MATERIAL

## Data Availability

If necessary, we are willing to share the de-identified participant data of individuals, that is, the feature tables of all cases extracted in this study, including all deep learning and radiomics features, as well as the clinical feature tables of all cases. Please contact the corresponding author for access. The data is proprietary and is only for the purpose of reproducing the research results. Secondary use without permission is prohibited.

## Abbreviations

AIC: Akaike information criterion
AUC: Area under the curve
c_LNM: Clinically suspected lymph node metastasis
CE-T1WI: Contrast-enhanced T1-weighted imaging
DCA: Decision curve analysis
DL: Deep learning
DLRC: Deep learning-radiomics-clinical fusion model
DWI: Diffusion-weighted imaging
FIGO: The International Federation of Gynecology and Obstetrics
FS-T2WI: Fat-saturated T2-weighted imaging
ICCs: Intra- and interclass correlation coefficients
LASSO: Least absolute shrinkage and selection operator
LNM: Lymph node metastasis
LNSD: Lymph node short-axis diameter
mp-MRI: Multiparametric-MRI
NPV: Negative predictive value
PPV: Positive predictive value
ROC: Receiver operating characteristic
ROI: Region of interest
SRA: Standardized radiological assessment
VOI: Volume of interest
