# Supplementary material for: Primary tumor-derived, multiparametric MRI-based deep learning-radiomics-clinical model for predicting lymph node metastasis in early-stage cervical cancer
Source: Insights Imaging. 2026 Feb 9;17:38. doi: 10.1186/s13244-026-02211-w (PMC12886668; doi:10.1186/s13244-026-02211-w)
Supplement: Supplementary file 1 — ELECTRONIC SUPPLEMENTARY MATERIAL [file 13244_2026_2211_MOESM1_ESM.pdf]

Primary Tumor-derived, Multiparametric MRI-based Deep Learning-Radiomics-Clinical Model for Predicting Lymph Node Metastasis in Early-stage Cervical Cancer

ELECTRONIC SUPPLEMENTARY MATERIAL

Supplementary Method S1

The independent lymph node assessments and measurements by Radiologist 1 (Y.H.B., 5 years of experience) and Radiologist 2 (Y.A.L., 11 years of experience), along with the final consensus readings, are provided in Supplementary file 1.

The measurement protocol was as follows: (1) Measurements were recorded to the nearest 0.1 mm. To account for inherent measurement variability in clinical practice, a discrepancy of less than 1.0 mm between the two radiologists was resolved by averaging their measurements. (2) For any discrepancy of 1.0 mm or greater, or for any disagreement in qualitative assessment, the case was adjudicated by a senior radiologist (J.W.Q., 20 years of experience) to achieve a final consensus. (3) For lymph nodes with a short-axis diameter (LNSD) between 8 mm and 10 mm, the long-axis diameter was also measured to calculate the short-to-long axis ratio. Disagreements in ratio assessment were likewise resolved by the senior radiologist.

Supplementary Method S2

The contrast agent used for enhanced scanning was gadopentetate dimeglumine (Magnevist, Bayer, Germany), administered at a dose of 0.1 or 0.2 mmol/kg with an injection rate of 2 ml/s. Venous-phase images were acquired 90–150 seconds after gadolinium injection. To minimize motion artifacts caused by bowel peristalsis, patients were required to fast for 4–6 hours before MRI scanning.

Supplementary Method S3

Feature extraction was implemented in Windows 11 with Python 3.9 using PyTorch 2.0 (https://pytorch.org) and executed on an Nvidia RTX 4090 workstation.

Image preprocessing was performed following these steps for radiomics feature extraction: (1) resampling voxel size to 1×1×1 mm^3; (2) Z-score normalization; (3) gray-level discretization into five intensity levels. Radiomics features were extracted from three raw segmented image sequences and their filtered versions using PyRadiomics (version 3.1.0, www.radiomics.io/pyradiomics.html).

Feature Categories: morphological: 3D shape (14 features)； first-order statistics: histogram-based features (18 features)； texture features: 1) Gray Level Co-occurrence Matrix (GLCM, 24 features); 2) Gray Level Run Length Matrix (GLRLM, 16 features);3) Gray Level Size Zone Matrix (GLSZM, 16

features); 4) Gray Level Dependence Matrix (GLDM, 14 features); 5) Neighbouring Gray Tone Difference Matrix (NGTDM, 5 features).

Feature extraction sources: original image: morphological, first-order, and texture features (107 features); Laplacian of Gaussian (LoG, $\sigma = 3, 5$): first-order and texture features (186 features); wavelet transform (8 filter parameters): first-order and texture features (744 features); square root filter: first-order and texture features (93 features); logarithm filter: first-order and texture features (93 features); exponential filter: first-order and texture features (93 features); gradient filter: first-order and texture features (93 features); Local Binary Pattern (LBP): three parameters (k, m1, m2): first-order and texture features (279 features).

With three sequences (DWI, FS-T2WI, and CE-T1WI), a total of 5064 radiomic features were extracted.

Images underwent the following preprocessing steps for DL features extraction: (1) voxel resampling to $1\times1\times1$ mm^3; (2) Z-score normalization. The entire network consists of multiple residual blocks stacked together, divided into several stages, where the feature map size is halved and the number of channels is doubled at each stage. The fully connected layer was removed, and DL features were extracted from the penultimate layer of the modified ResNet models. For 2D analysis, axial slices demonstrating the maximal tumor were selected. Following standardized preprocessing, a 224×224 mm bounding box encompassing the entire tumor ROI was extracted for 2D convolutional feature extraction. For 3D image feature extraction, a $224\times224\times224$ mm cube containing

the entire tumor region was cropped and used as the input for the 3D model to extract 3D DL features.

Preprocessing of radiomics and DL features was performed: Z-score normalization was applied to the training cohort, and the same normalization parameters were used for the internal and external validation cohorts. Feature selection was performed exclusively within the training cohort to ensure the independence of the internal and external validation cohorts.

Supplementary Figures

Figure S1. Schematic diagram of ROI delineation on FS-T2WI, DWI, and CE-T1WI

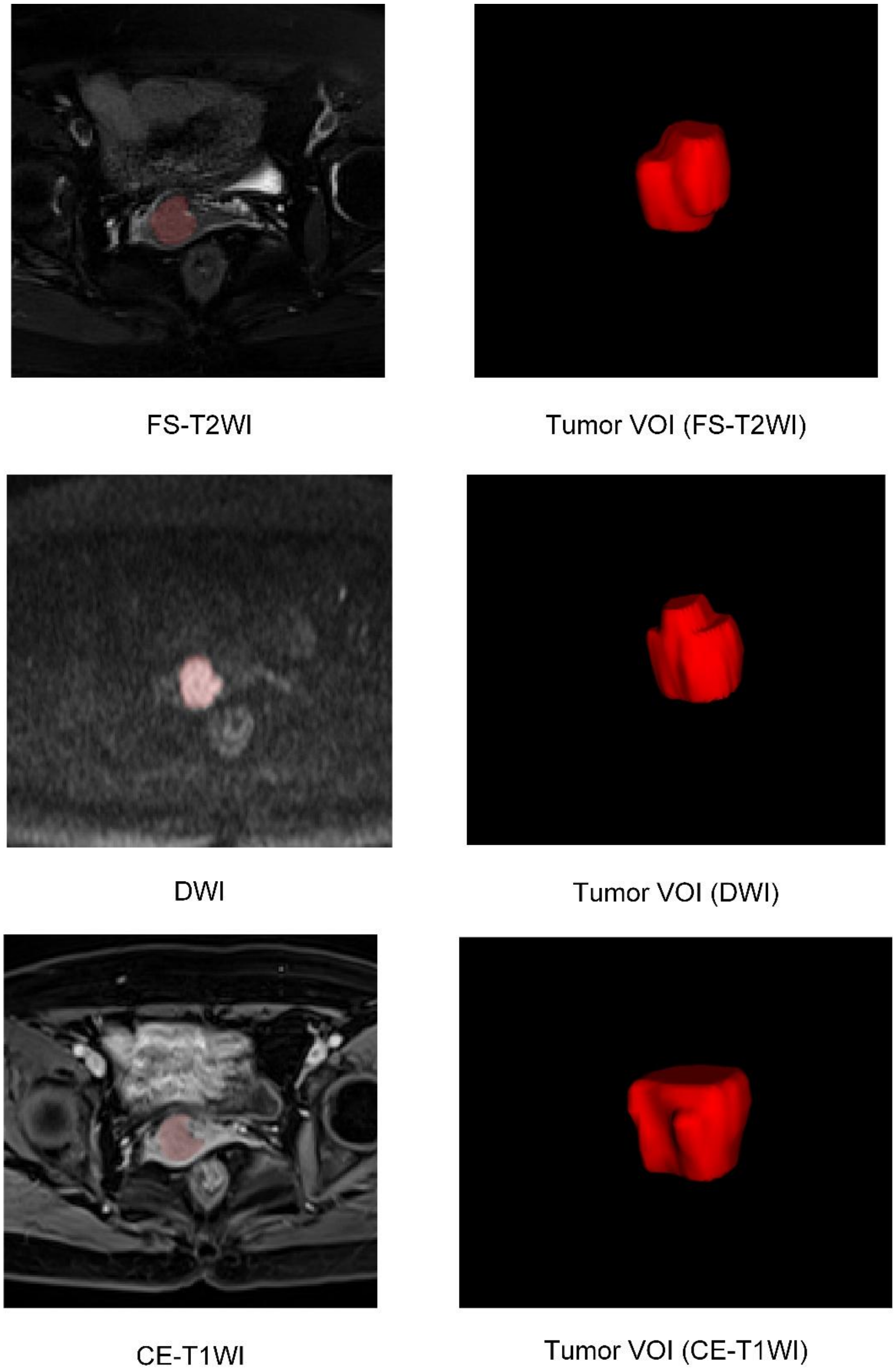

(FS-T2WI: fat-saturation T2-weighted imaging; DWI: diffusion weighted imaging; CE-T1WI: contrast-enhanced T1-weighted imaging)

Figure S2. Principal component analysis scatterplots of radiomics features before and after ComBat harmonization

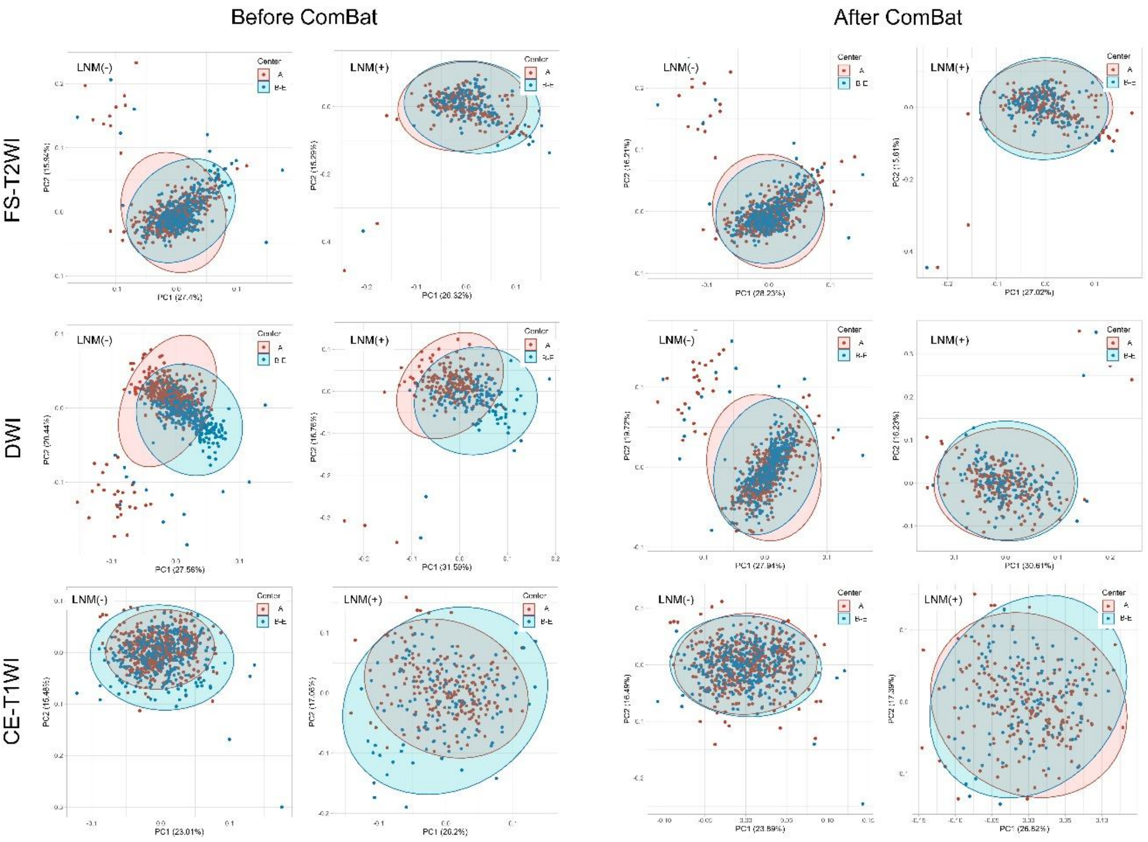

(Two-dimensional scatterplots visualize the first two principal components of radiomics features used for modeling from Center A and Centers B-E before and after ComBat harmonization. The proportions of the two principal components are displayed on the X- and Y-axes, respectively. Data from Center A and Centers B-E are represented in red and blue, respectively.)

Figure S3. Principal component analysis scatterplots of ResNet152_2D features before and after ComBat harmonization

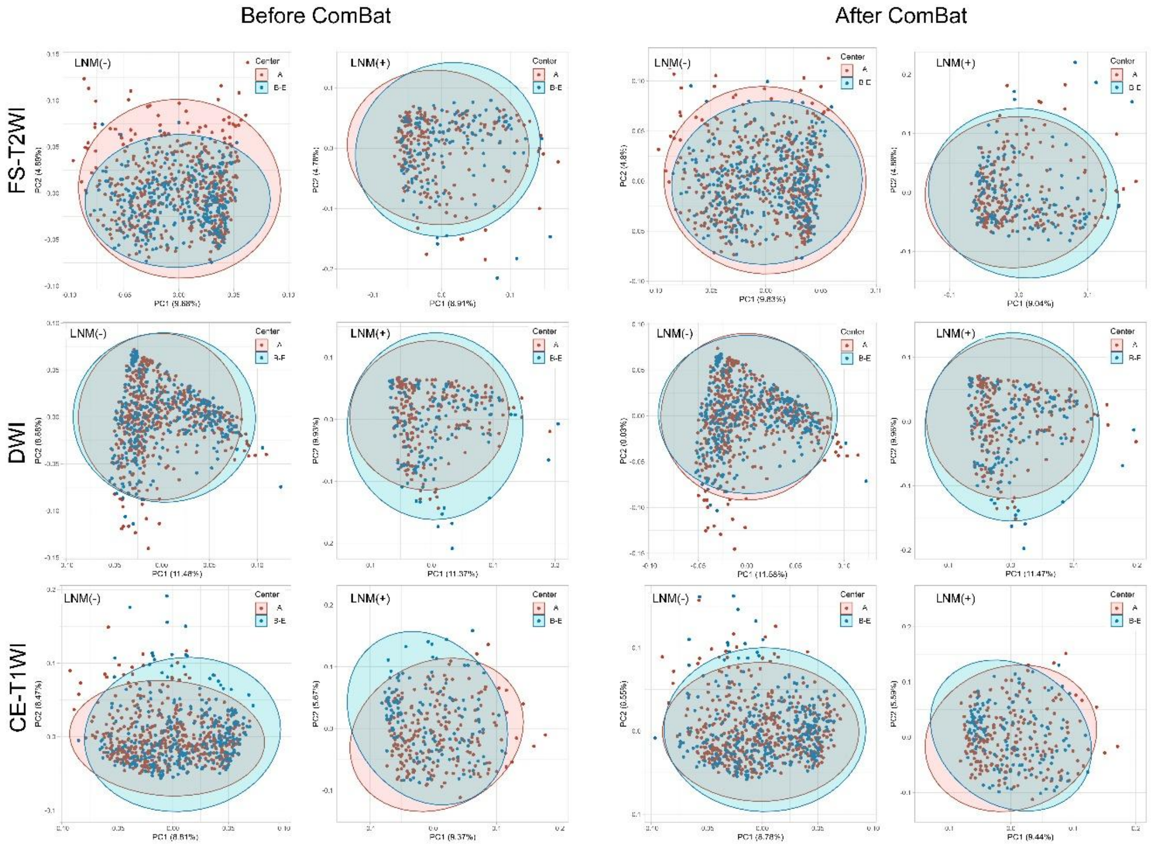

(Two-dimensional scatterplots visualize the first two principal components of ResNet152_2D features used for modeling from Center A and Centers B-E before and after ComBat harmonization. The proportions of the two principal components are displayed on the X- and Y-axes, respectively. Data from Center A and Centers B-E are represented in red and blue, respectively.)

Figure S4. Violin plots of radiomics feature distributions in the LNM-negative and LNM-positive subgroups in the training cohort

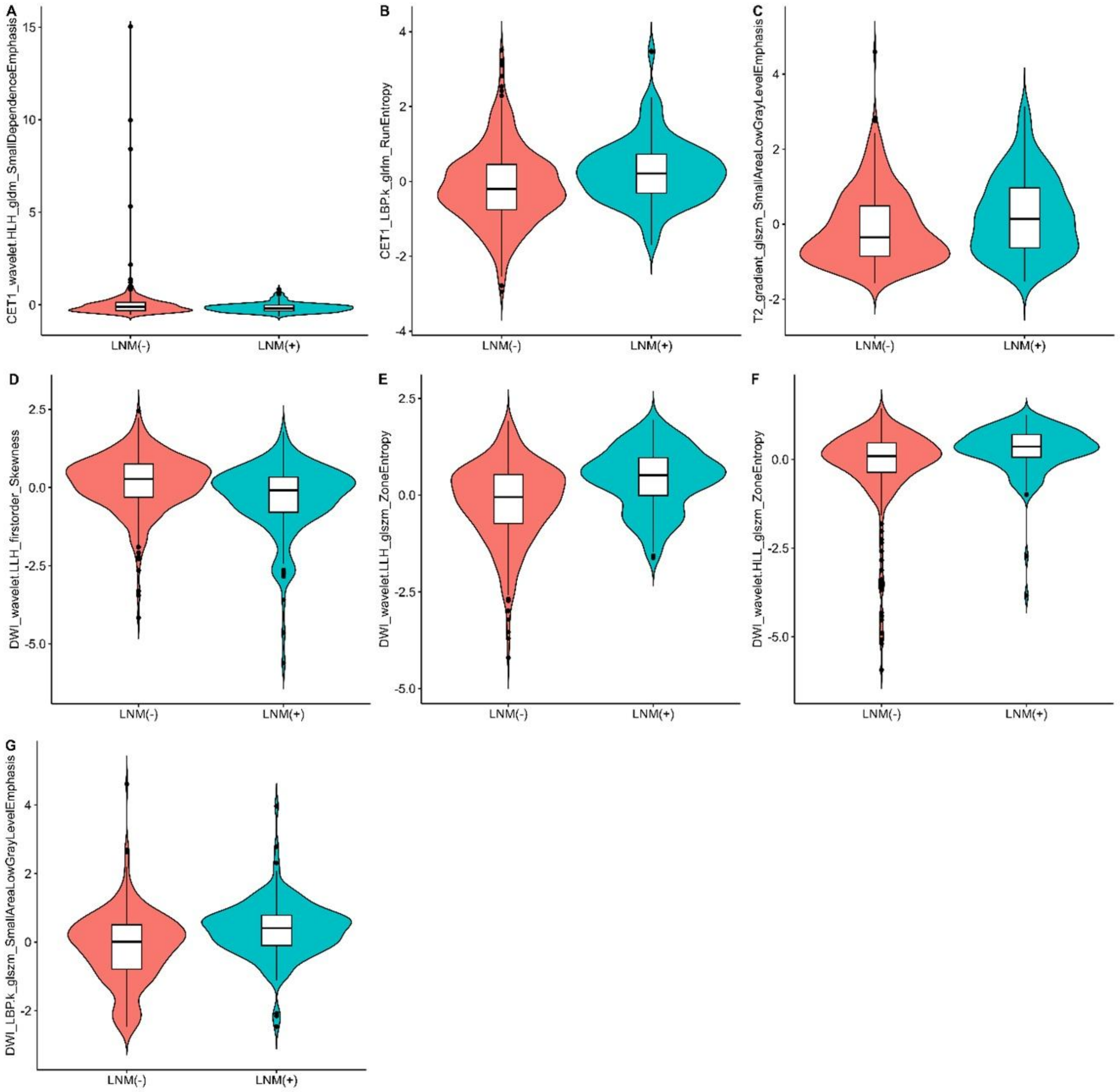

(The distributions of the seven radiomics features retained after multivariable logistic regression analysis are shown, including four wavelet features, two local binary pattern (LBP) features, and one gradient filtering feature. The Y-axis represents the normalized feature value distributions.)

Figure S5. Heatmap of radiomics feature correlations in the training cohort

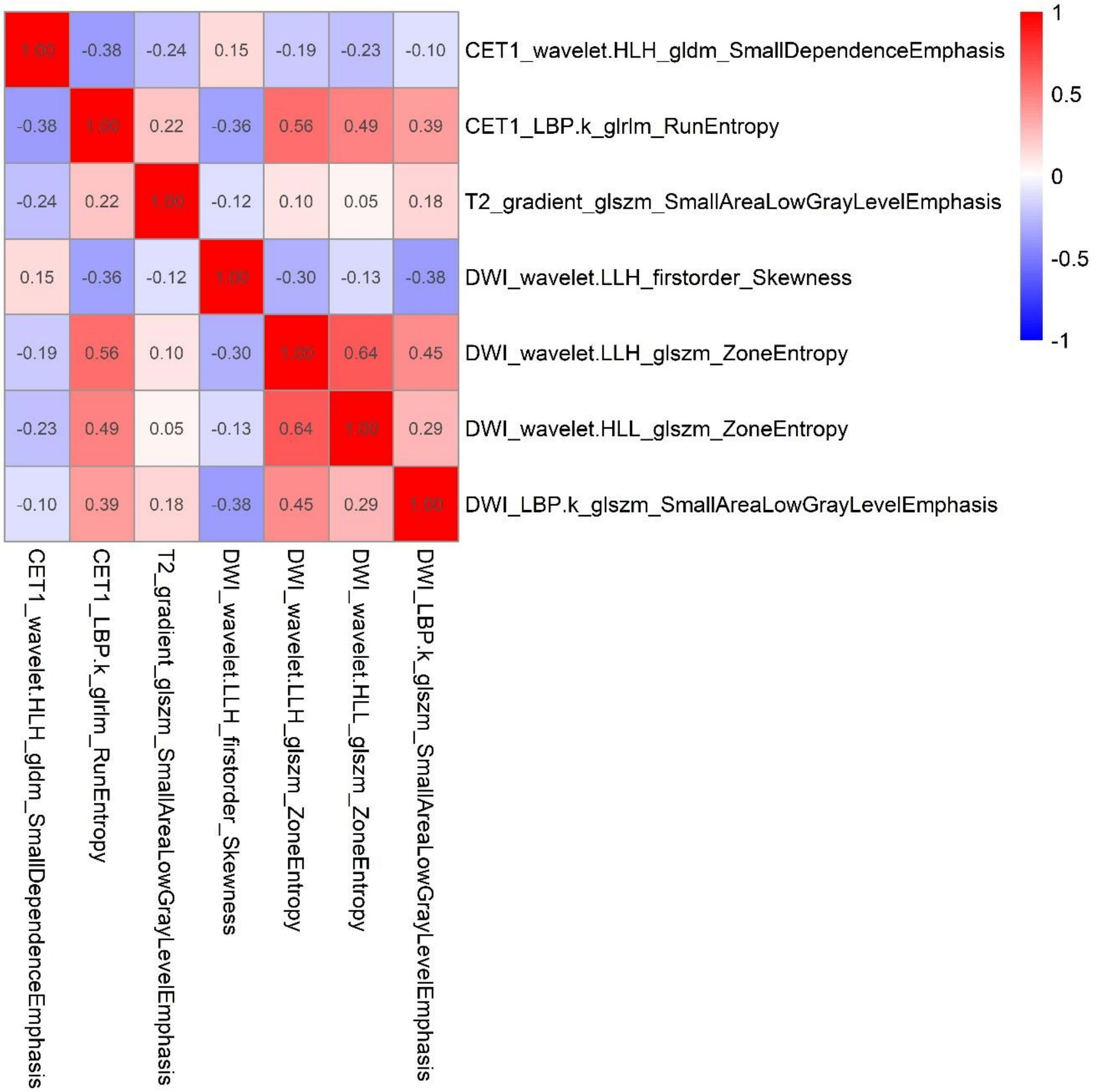

Figure S6. Violin plots of ResNet152_2D feature distributions in the LNM-negative and LNM-positive subgroups in the training cohort

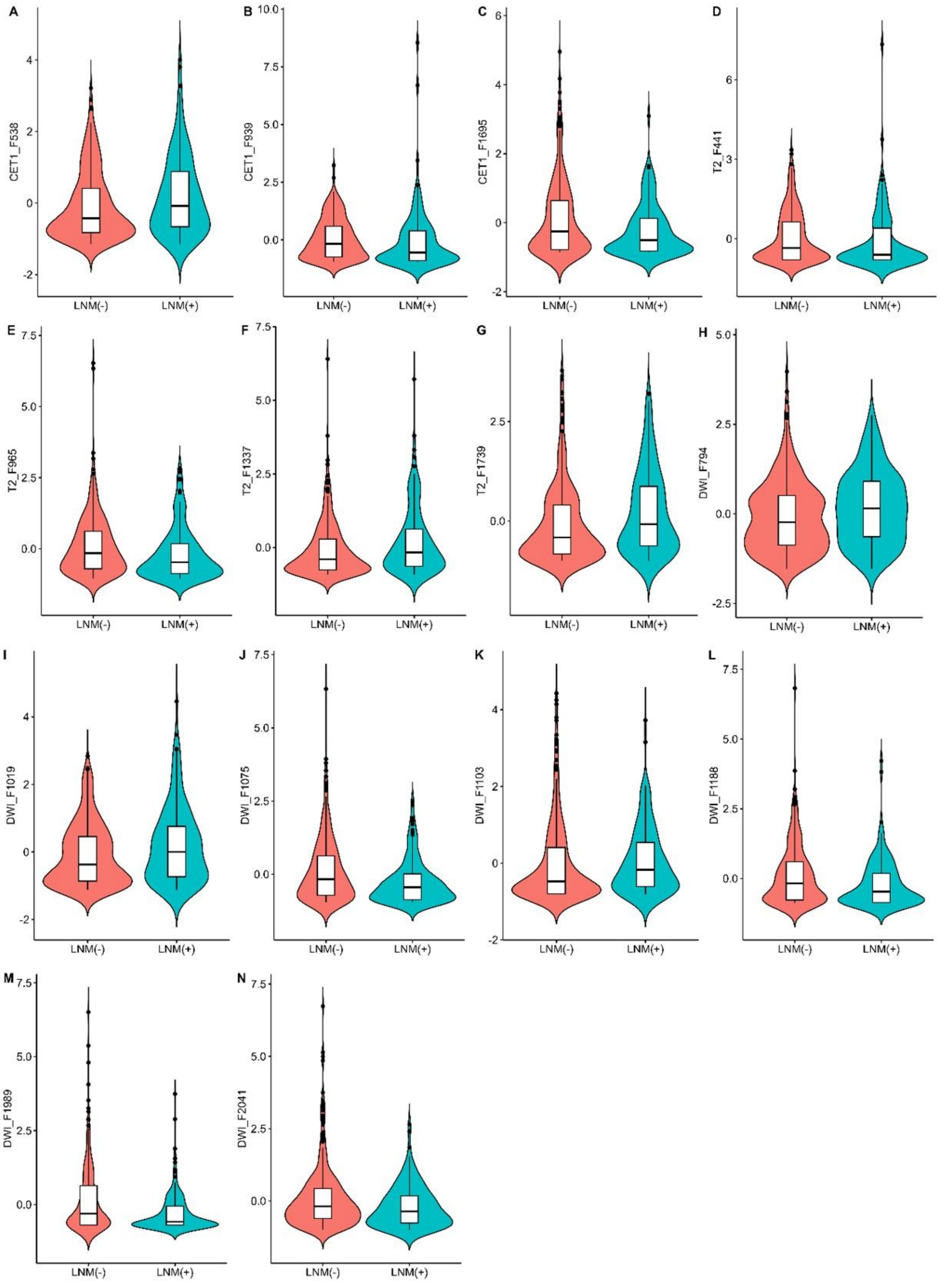

(The distributions of the 14 DL features retained after multivariable logistic regression are shown. The Y-axis represents the normalized feature value distributions.)

Figure S7. Heatmap of ResNet152_2D feature correlations in the training cohort

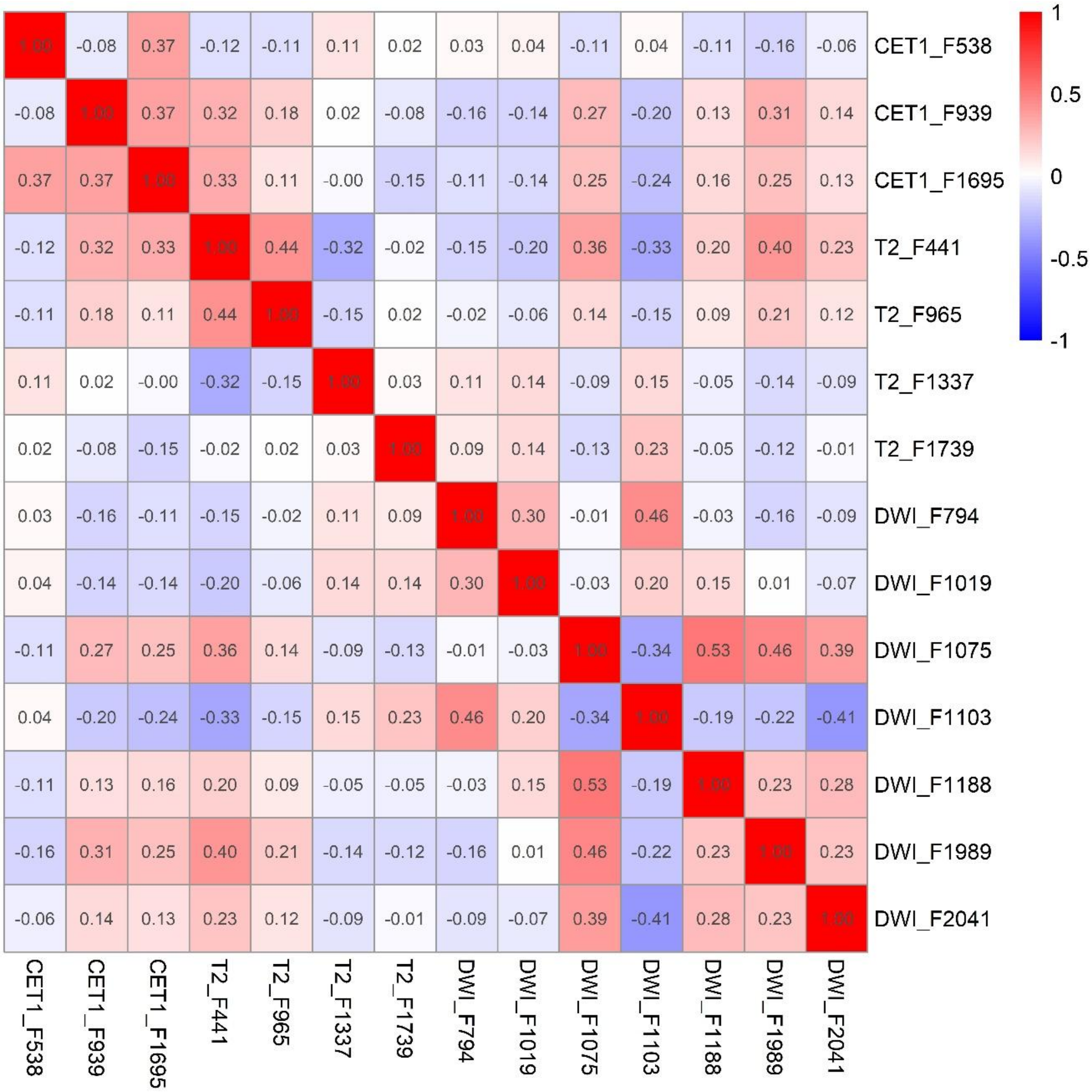

	CET1_F538	CET1_F939	CET1_F1695	T2_F441	T2_F965	T2_F1337	T2_F1739	DWI_F794	DWI_F1019	DWI_F1075	DWI_F1103	DWI_F1188	DWI_F1989	DWI_F2041
CET1_F538	1.00	-0.08	0.37	-0.12	-0.11	0.11	0.02	0.03	0.04	-0.11	0.04	-0.11	-0.16	-0.06
CET1_F939	-0.08	1.00	0.37	0.32	0.18	0.02	-0.08	-0.16	-0.14	0.27	-0.20	0.13	0.31	0.14
CET1_F1695	0.37	0.37	1.00	0.33	0.11	-0.00	-0.15	-0.11	-0.14	0.25	-0.24	0.16	0.25	0.13
T2_F441	-0.12	0.32	0.33	1.00	0.44	-0.32	-0.02	-0.15	-0.20	0.36	-0.33	0.20	0.40	0.23
T2_F965	-0.11	0.18	0.11	0.44	1.00	-0.15	0.02	-0.02	-0.06	0.14	-0.15	0.09	0.21	0.12
T2_F1337	0.11	0.02	-0.00	-0.32	-0.15	1.00	0.03	0.11	0.14	-0.09	0.15	-0.05	-0.14	-0.09
T2_F1739	0.02	-0.08	-0.15	-0.02	0.02	0.03	1.00	0.09	0.14	-0.13	0.23	-0.05	-0.12	-0.01
DWI_F794	0.03	-0.16	-0.11	-0.15	-0.02	0.11	0.09	1.00	0.30	-0.01	0.46	-0.03	-0.16	-0.09
DWI_F1019	0.04	-0.14	-0.14	-0.20	-0.06	0.14	0.14	0.30	1.00	-0.03	0.20	0.15	0.01	-0.07
DWI_F1075	-0.11	0.27	0.25	0.36	0.14	-0.09	-0.13	-0.01	-0.03	1.00	-0.34	0.53	0.46	0.39
DWI_F1103	0.04	-0.20	-0.24	-0.33	-0.15	0.15	0.23	0.46	0.20	-0.34	1.00	-0.19	-0.22	-0.41
DWI_F1188	-0.11	0.13	0.16	0.20	0.09	-0.05	-0.05	-0.03	0.15	0.53	-0.19	1.00	0.23	0.28
DWI_F1989	-0.16	0.31	0.25	0.40	0.21	-0.14	-0.12	-0.16	0.01	0.46	-0.22	0.23	1.00	0.23
DWI_F2041	-0.06	0.14	0.13	0.23	0.12	-0.09	-0.01	-0.09	-0.07	0.39	-0.41	0.28	0.23	1.00

Supplementary Tables

Table S1. MRI Equipment, Scanning Protocols, and Parameters at Each Center

Table S1. MRI Equipment, Scanning Protocols, and Parameters at Each Center

Cen	MRI	NA	b-value s/mm²	seq	TR ms	TE ms	MAT	NEX	ST mm	GAP mm	FA
A	1.5T	T2		SE	4000-6500	83	256*256	1	5	2	150
	Avanto	DWI	1000	EPI	2400-3300	78-83	320*320	3	5	1.5-2	90
	Siemens	CET1		VIBE	4.9	2.4	256*256	2	4.5	0	10
B	3.0T	T2		FSE	4400-4800	79-82	512*512	1.1	5	1	111
	SIGNA Pioneer	DWI	800	EPI	7800-8100	58-59	256*256	5	5	1	90
	General Electric	CET1		LAVA	6.5	3.5	512*512	0.7	4	0	15
	3.0T	T2		FSE	5300-5600	98-104	512*512	1.3	5	1	111
	SIGNA Architect	DWI	800	EPI	5600-5900	69-70	256*256	4	5	1	90
	General Electric	CET1		LAVA	4.6	1.7	512*512	0.7	4	-2	15
	3.0T	T2		TSE	6500	104	640*640	2	7	1.4	180
	MAGNETOM Skyra	DWI	800	EPI	3700	70	256*256	16	6.5	1.3	90
	Siemens	CET1		VIBE	3.6	1.3	384*384	1	4.5	0	9
	3.0T	T2		TSE	3030	65	448*448	1	6	1.8	120
	MAGNETOM Prisma	DWI	800	EPI	5900	53	272*272	5	6	1.8	90
	Siemens	CET1		VIBE	3.4	1.3	640*640	1	3	0	11
	1.5T	T2		FSE	2600-3000	74-78	512*512	1.5	6	2	110
	Signa	DWI	800	EPI	5900-6400	71-72	256*256	6	6	2	90
	General Electric	CET1		LAVA	6.2	3.1	1024*1024	0.7	5	0	12
	1.5T	T2		FSE	3929	81.6	480*480	3	5	1	150
	uMR 588	DWI	800	EPI	3200	80.5	288*288	6	5	1	90

	United Imaging	CET1		QUICK 3D	4.7	2.1	512*512	1	3	0	10
	3.0T	T2		TSE	6080	104	320*320	1	7	1.4	180
	MAGNETOM Verio	DWI	800	EPI	5500	87	170*170	3	7	1.4	90
	Siemens	CET1		VIBE	3.6	1.3	384*384	1	4.5	0	9
**C**	1.5T	T2		FSE	2600-3000	60-61	512*512	1	6	2	160
	OPTIMA 360	DWI	800	EPI	3100-4000	73-76	256*256	6	6	2	90
	General Electric	CET1		LAVA	3.8	1.7	512*512	0.7	4.6	-2.3	15
**D**	1.5T	T2		TSE	4200-5200	82	640*640	2	4.5-5	0.9-1	162
	MAGNETOM Aera	DWI	800	EPI	5100	80	336*336	6	4	0.8	90
	Siemens	CET1		VIBE	4.5	2.2	640*640	1	3	0	10
**E**	3.0T	T2		FSE	4000	88.34	384*384	1.7	4	0.4	105
	uMR 780	DWI	800/1000	EPI	5001	71	256*256	6	4	0.4	90
	United Imaging	CET1		QUICK 3D	3.5	1.6	480*480	1	2	0	10
	3.0T	T2		SE	3000	64	320*320	1	5	1	160
	MAGNETOM Verio	DWI	800/1000	EPI	4400-5100	72-83	192*192	3-4	5	1	90
	Siemens	CET1		VIBE	3.9	1.9	320*320	1	3	0	9

Cen: center; NA: name; seq: sequence; TR: repetition time; TE: echo time; MAT: matrix; NEX: number of excitation; ST: slice thickness; FA: flip angle.

Table S2. Details of Features in the Radiomics Model, DL Model, and DLRC Fusion Model

model	Features' name	coefficient	*P*	OR
**Radiomics**				
	(Intercept)	-1.036	<0.001	
	CET1_wavelet.HLH_gldm_SmallDependenceEmphasis	-0.544	0.193	0.581
	CET1_LBP.k_glrlm_RunEntropy	-0.264	0.076	0.768
	T2_gradient_glszm_SmallAreaLowGrayLevelEmphasis	0.321	0.005	1.379
	DWI_wavelet.LLH_firstorder_Skewness	-0.361	0.003	0.697
	DWI_wavelet.LLH_glszm_ZoneEntropy	0.542	0.001	1.720
	DWI_wavelet.HLL_glszm_ZoneEntropy	0.297	0.126	1.345
	DWI_LBP.k_glszm_SmallAreaLowGrayLevelEmphasis	0.265	0.053	1.303
**ResNet50_3D**				
	(Intercept)	-0.952	<0.001	
	CET1_F177	-0.542	<0.001	0.582
	CET1_F593	-0.223	0.047	0.800
	CET1_F1473	0.195	0.099	1.215
	CET1_F2014	0.292	0.012	1.339
	T2_F1956	0.211	0.042	1.235
	DWI_F1089	0.297	0.006	1.346
	DWI_F1738	-0.212	0.065	0.809
**ResNet101_3D**				
	(Intercept)	-0.951	<0.001	
	CET1_F1419	0.352	0.004	1.422
	CET1_F1519	-0.252	0.023	0.777
	CET1_F2038	-0.165	0.133	0.848
	T2_F1318	-0.211	0.054	0.810
	DWI_F160	1.006	0.037	2.734
	DWI_F1519	-0.257	0.042	0.773
**ResNet152_3D**				
	(Intercept)	-0.901	<0.001	
	CET1_F348	-0.421	0.001	0.656

	CET1_F527	-0.341	0.005	0.711
	CET1_F952	-0.348	0.002	0.706
	CET1_F999	-0.369	0.015	0.692
	CET1_F1278	0.367	0.002	1.444
	CET1_F1528	-0.322	0.003	0.724
	T2_F358	0.242	0.024	1.274
**ResNet50_2D**				
	(Intercept)	-0.967	<0.001	
	CET1_F296	-0.211	0.091	0.810
	CET1_F768	0.271	0.010	1.312
	CET1_F1150	0.211	0.049	1.235
	T2_F671	-0.228	0.052	0.796
	T2_F1292	0.175	0.111	1.191
	T2_F1625	0.340	0.002	1.405
	DWI_F652	0.307	0.004	1.360
	DWI_F1849	0.378	0.001	1.460
**ResNet101_2D**				
	(Intercept)	-1.018	<0.001	
	CET1_F44	-0.400	0.003	0.670
	CET1_F824	-0.558	0.002	0.572
	CET1_F918	-0.352	0.022	0.704
	CET1_F1154	0.250	0.021	1.284
	CET1_F1618	-0.282	0.044	0.755
	T2_F613	0.262	0.019	1.299
	T2_F882	-0.246	0.047	0.782
	T2_F1163	0.223	0.038	1.250
	DWI_F750	-0.207	0.115	0.813
	DWI_F1420	0.285	0.008	1.329
**ResNet152_2D**				
	(Intercept)	-0.999	<0.001	
	CET1_F538	0.367	0.003	1.444
	CET1_F939	0.207	0.074	1.231
	CET1_F1695	-0.535	<0.001	0.586
	T2_F441	0.372	0.008	1.451
	T2_F965	-0.319	0.014	0.727
	T2_F1337	0.246	0.026	1.279
	T2_F1739	0.258	0.015	1.294
	DWI_F794	0.224	0.069	1.251
	DWI_F1019	0.246	0.031	1.279

	DWI_F1075	-0.316	0.032	0.729
	DWI_F1103	-0.352	0.008	0.704
	DWI_F1188	-0.206	0.119	0.814
	DWI_F1989	-0.360	0.025	0.697
	DWI_F2041	-0.254	0.057	0.776
**DLRC**				
	(Intercept)	-5.271	<0.001	
	c_LNM	2.133	<0.001	8.438
	Rad_score	2.999	<0.001	20.073
	DL_score	3.326	<0.001	27.833

OR: odds ratio.
